# Tryptophan 2,3-Dioxygenase-2 in Uterine Leiomyoma: Dysregulation by MED12 Mutation Status

**DOI:** 10.1007/s43032-022-00852-y

**Published:** 2022-01-21

**Authors:** Anne P. Hutchinson, Ping Yin, Ilona Neale, John S. Coon, Stacy A. Kujawa, Shimeng Liu, Serdar E. Bulun

**Affiliations:** 1 Department of Obstetrics and Gynecology, Division of Reproductive Science in Medicine, Feinberg School of Medicine, Prentice Women’s Hospital, Northwestern University, 250 E. Superior Street , Chicago, IL 60611 USA; 2 Department of Medical Oncology, Dana-Farber Cancer Institute, Boston, MA USA

**Keywords:** Uterine leiomyoma, TDO2, MED12, Tryptophan, Progesterone, Kynurenine

## Abstract

Uterine leiomyomas (fibroids) are common benign tumors in women. The tryptophan metabolism through the kynurenine pathway plays important roles in tumorigenesis in general. Leiomyomas expressing mutated mediator complex subunit 12 (mut-MED12) were reported to contain significantly decreased tryptophan levels; the underlying mechanism and the role of the tryptophan metabolism-kynurenine pathway in leiomyoma tumorigenesis, however, remain unknown. We here assessed the expression and regulation of the key enzymes that metabolize tryptophan. Among these, the tissue mRNA levels of tryptophan 2,3-dioxygenase (TDO2), the rate limiting enzyme of tryptophan metabolism through the kynurenine pathway, was 36-fold higher in mut-MED12 compared to adjacent myometrium (*P* < 0.0001), and 14-fold higher compared to wild type (wt)-MED12 leiomyoma (*P* < 0.05). The mRNA levels of other tryptophan metabolizing enzymes, IDO1 and IDO2, were low and not significantly different, suggesting that TDO2 is the key enzyme responsible for reduced tryptophan levels in mut-MED12 leiomyoma. R5020 and medroxyprogesterone acetate (MPA), two progesterone agonists, regulated TDO2 gene expression in primary myometrial and leiomyoma cells expressing wt-MED12; however, this effect was absent or blunted in leiomyoma cells expressing G44D mut-MED12. These data suggest that MED12 mutation may alter progesterone-mediated TDO2 expression in leiomyoma, leading to lower levels of tryptophan in mut-MED12 leiomyoma. This highlights that fibroids can vary widely in their response to progesterone as a result of mutation status and provides some insight for understanding the effect of tryptophan-kynurenine pathway on leiomyoma tumorigenesis and identifying targeted interventions for fibroids based on their distinct molecular signatures.

**Supplementary Information:**

The online version contains supplementary material available at 10.1007/s43032-022-00852-y.

## Introduction

Uterine leiomyomas (fibroids, LM) are benign neoplasms that arise from uterine smooth muscle and represent the most common benign tumor in reproductive-age women. While a majority of LM are asymptomatic, they commonly cause menorrhagia, dysmenorrhea, and infertility and approximately 30% of identified fibroids require intervention [1]. While some medical therapies have shown promise in symptomatic management, hysterectomy or myomectomy remains the most common approach to treatment. Advances in minimally invasive surgery have improved recovery and minimized hospital stays overall for these patients, but the socioeconomic burden remains significant, costing an estimated $34.4 billion for the 200,000 hysterectomies and 30,000 myomectomies performed each year to treat fibroids [2]. New approaches to medical management for fibroids are desperately needed.

With few exceptions, LM originate from somatic mutations in myometrium (MM) cells, resulting in progressive loss of growth regulation leading to unchecked growth. A variety of mutations have been identified that seem to give rise to unique growth patterns [3]. One distinct subtype carries a mutation in mediator complex subunit 12 (mut-MED12) and comprises 70% of all LM. The most common mutation within this family is a single point mutation at codon 44 in exon 2 of the MED12 gene, the G44D mutation [4]. MED12 mutation leads to a configurational change that alters its interaction with transcriptional co-activator pathway proteins including cyclin C, leading to a loss in CDK activity [5].

Global metabolomics profiling of LM showed decreased levels of tryptophan in mut-MED12 LM tissue compared to tumors expressing wild type (wt)-MED12 [6]. Three enzymes (tryptophan 2,3-dioxygenase [*TDO2*], indoleamine-2,3-dioxygenease 1 [IDO1], and IDO2) catalyze the oxidation of L-tryptophan to N-formyl-L-kynurenine, as the first and rate-limiting step of kynurenine production, which plays important roles in tumorigenesis [7–10]. However, the mechanisms leading to decreased tryptophan in mut-MED12 LM, and the role of the tryptophan-kynurenine pathway in LM tumorigenesis, remain unclear.

Estrogen and progesterone driving LM cell proliferation, survival, and extracellular matrix formation [11]. Progesterone regulates TDO2 expression in endometrium and breast tissue, contributing to both normal tissue function and tumor growth [12, 13]. To better understand the role of the tryptophan-kynurenine pathway in LM, with the ultimate goal of developing new therapeutics for LM, here we examined the effect of MED12 mutation on tryptophan metabolism. We tested the hypothesis that progestins regulate TDO2 in LM and MM tissues and that MED12 mutation impairs progestin-regulated TDO2 expression, leading to a decrease in tryptophan in mut-MED12 LM.

## Materials and Methods

### Tissue Collection

The study was approved by the Northwestern University Institutional Review Board, and informed consent was obtained from all participants for collection and use of LM and MM tissues (Reproductive Tissue Registry STU00018080). All tissues were obtained from premenopausal women undergoing either myomectomy or hysterectomy (age 47 ± 4 years, range 42–54 years). Patients receiving hormone treatment 6 months prior to surgery were excluded. Matched LM and MM tissues were collected from each patient and underwent complete pathologic assessment prior to use in experiments.

### Primary Cell Culture and Treatment

Tissues were dissociated and cells were isolated as previously described [14]. Briefly, tissues were rinsed with sterile PBS, minced finely, and digested with collagenase (#C0130-1G, Sigma, St. Louis, MO) and DNase (#D5025-150KU, Sigma) at 37 °C. Cells were strained, rinsed, and suspended in Smooth Muscle Cell Growth Medium (#CC-3182, Lonza, Basel Switzerland) supplemented with 5% fetal bovine serum, insulin, hFGF-B, GA-1000, hEGF, and 1% antibiotic/antimycotic in a humidified atmosphere, with 5% CO_2_ at 37 °C. All cells were used within two passages after initial culture. Cells were grown to 80% confluence and serum starved overnight before treatment with vehicle (0.1% ethanol), R5020 (#50–905-1338, Thermo Fisher Scientific, Waltham, MA), or medroxyprogesterone acetate (MPA) (#1,378,001-200MG, Sigma) at different doses (10^–8^, 10^–7^, 10^–6^, and 10^–5^ M) for 6, 24, 48, and 72 h.

### Real-Time Quantitative PCR

Total RNA from LM and matched MM tissue or cultured primary cells was extracted using the RNeasy mini kit (#74,106, QIAGEN, Germantown, MD). cDNA was synthesized using qScript cDNA Supermix (#95,048–100, VWR International, Radnor, PA). mRNA levels of TDO2, IDO1, and IDO2 were quantified using quantitative real-time PCR (RT-qPCR) with Taqman Universal Mastermix (#4,364,338, Thermo Fisher Scientific) and normalized to TATA-BOX Binding Protein (TBP). Primers used were purchased from Integrated DNA Technologies (TDO2: Hs.PT.58.3092178; IDO1: Hs.PT.58.9247; IDO2: Hs.PT.58.3013208) and Thermo Fisher Scientific (TBP: Hs00427260_m1). RT-qPCR was performed on a QuantStudio 12 K Flex instrument (Thermo Fisher Scientific). Expression levels were calculated by applying the comparative cycle threshold (Ct) method. All experiments were carried out with a non-template control.

### Immunoblot Analysis

Total protein was extracted from primary LM or MM cells using RIPA buffer and quantified using BCA assay (23,225; Thermo Fisher Scientific) per the manufacturer’s protocol. Protein was then diluted in 4X LDS sample buffer (NP0007; Thermo Fisher Scientific), electrophoresed on a 4% to 12% Novex Bis–Tris polyacrylamide precast gel (NP0321BOX; Thermo Fisher Scientific), and transferred onto polyvinylidene difluoride membrane. The membranes were incubated with primary antibody against TDO2 (15,880–1-A, Proteintech) at 4 °C in 5% nonfat milk overnight, followed by incubation with HRP-linked anti-rabbit IgG (7074S, Cell Signaling Technology) for 1 h at room temperature. β-actin (HRP-60008, Proteintech) was used as loading control. Detection was performed using Luminata Crescendo horseradish peroxidase substrate (WBLUR0100; Millipore).

### Genotyping

MED12 mutation screening was performed by Sanger sequencing using genomic DNA isolated from snap-frozen LM and MM tissues. The MED12 mutation status in primary cell cultures was confirmed using cDNA isolated from cultured cells. Genomic DNA or cDNA was amplified using a hot start DNA polymerase kit (#71,086–3, Sigma) and primers as previously described [4], followed by sequencing at Northwestern University Sanger Sequencing Core. Recent high throughput sequencing studies have revealed recurrent and mutually exclusive driver mutations in LM: including MED12 mutations, high mobility group AT-hook 2 (HMGA2) rearrangements, biallelic inactivation of fumarate hydratase (FH), and collagen, type IV, alpha 5 and collagen, type IV, alpha 6 (COL4A5-COL4A6) deletions [15]. LM are usually categorized into different subtypes based on their gene mutation signature. In this study, we termed LM that did not express mutant MED12 as wild type (wt)-MED12 LM.

### Statistical Analysis

Each experiment was conducted utilizing cells from at least three patients run in triplicate, followed by statistical analysis. All data are expressed as the mean ± standard error of mean (SEM). *P* values were calculated using Student’s *t* test (to compare two groups) or one-way ANOVA followed by Dunnett’s multiple comparison test (to compare three or more groups) using the GraphPad Prism software (GraphPad Inc., San Diego, CA). Differences were considered statistically significant when *P* < 0.05.

## Results

### TDO2 Gene Expression Is Upregulated in Leiomyoma Expressing mut-MED12

**Fig. 1:**
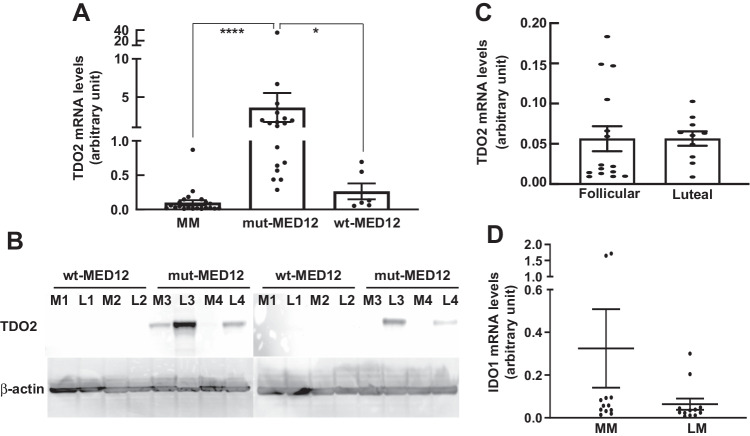
TDO2 gene expression is upregulated in leiomyoma expressing mut-MED12. Real-time qPCR quantification of TDO2 and IDO1 gene expression in LM carrying wt-MED12, LM carrying mut-MED12, and matched myometrium (MM) tissues. All values were normalized to TBP. **A** TDO2 expression was significantly higher in mut-MED12 LM (*n* = 18 patients) vs wt-MED12 LM (*n* = 6 patients) and MM (*n* = 24 patients). *P < 0.05, ****P < 0.0001. **B** TDO2 protein levels of primary LM (L) cells expressing mut-MED12 (*n* = 4 patients), LM cells expressing wt-MED12 (*n* = 4 patients), and their matched normal MM (M, *n* = 8 patients) cells were measured by western blot. β-actin was used as a loading control. **C**: Real-time qPCR quantification of TDO2 gene expression in MM tissues during the follicular (*n* = 15) and luteal (*n* = 10) phases of menstrual cycle. All values were normalized to TBP. **D**: IDO1 expression was not significantly different in LM and MM (*n* = 12 patients)

Based on a previous study showing different levels of tryptophan in mut-MED12 and wt-MED12 LM [6], we examined the gene expression levels of three enzymes involved in tryptophan metabolism through kynurenine pathway, TDO2, IDO1, and IDO2, by RT-qPCR in wt-MED12 LM (*n* = 6), mut-MED12 LM (*n* = 18), and matched MM tissues (*n* = 24). As shown in Fig. 1A, TDO2 expression was significantly higher in mut-MED12 LM compared to MM (35.96-fold, *P* < 0.0001) and wt-MED12 LM (13.66-fold, *P* < 0.05). We observed a trend of increased TDO2 expression in wt-MED12 LM vs MM tissues (2.6-fold), but it did not reach statistical significance. Western blot analysis confirmed that TDO2 protein level was also higher in mut-MED12 LM (Fig. 1B). We also evaluated the difference of the TDO2 expression levels in MM tissues between follicular (*n* = 15) and luteal (*n* = 10) phases of menstrual cycle and did not find significant change (Fig. 1C). IDO1 mRNA expression in LM and matched MM was low and not significantly different between LM and matched MM (Fig. 1D). Therefore, we compared IDO1 expression in LM vs their matched MM without separating LM into different genotypes. IDO2 mRNA expression was low and undetectable by RT-qPCR in both tissues (data not shown). These data suggest that dysregulation of TDO2 is responsible for the previously observed lower levels of tryptophan in LM carrying the MED12 mutation [6].

### Progestins Inhibit TDO2 Gene Expression in wt-MED12 Leiomyoma Cells

**Fig. 2:**
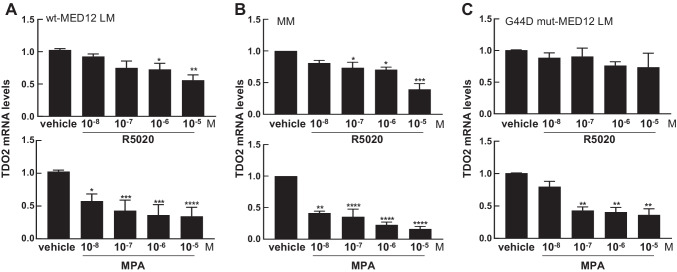
Progesterone agonists regulate TDO2 gene expression in LM and MM cells. Real-time qPCR quantification of TDO2 mRNA levels in primary cultured wt-MED12 LM cells (**A**), MM cells (**B**), and a mixture of cells expressing wt-MED12 and those expressing G44D mut-MED12 (**C**), after treatment with increasing doses (10^–8^, 10^–7^, 10^–6^, and 10^–5^ M) of R5020 (upper panels) and MPA (lower panels) for 24 h. All values were normalized to TBP and compared to vehicle. *P < 0.05, **P < 0.01, ***P < 0.005, ****P < 0.0001

Previous studies have reported the regulation of TDO2 by progesterone and its receptor (PR) in endometrial and breast cancer cells [12, 13], leading us to evaluate the effect of progestins on TDO2 expression in wt-MED12 LM cells. We treated primary cultures of wt-MED12 LM cells with increasing doses (10^–8^, 10^–7^, 10^–6^, and 10^–5^ M) of two progesterone agonists, R5020 and MPA, for 24 h. Each experiment was performed in triplicate in tissues from three unique patients. As shown in Fig. 2A upper panel, TDO2 gene expression decreased with increasing doses of R5020, with significant decreases at 10^–6^ and 10^–5^ M compared with vehicle-treated cells. The greatest reduction was observed at 10^–5^ M of R5020 (0.56 ± 0.09 of control, *P* < 0.01). MPA treatment also dose-dependently inhibited TDO2 expression, with a significant decrease at 10^–8^ M MPA to 0.58 ± 0.1 of the control level (*P* < 0.05; Fig. 2A lower panel). Maximal inhibition was observed at 10^–6^ M (TDO2 expression 0.36 ± 0.11 of control level [*P* = 0.001]). Sanger sequencing of genomic DNA isolated from tissue (data not shown) and cDNA isolated from treated cells from 3 patient samples revealed a pure wt-MED12 genotype (Supplemental Fig. 1A).

### Progestins Inhibit TDO2 Gene Expression in Myometrial Cells

We also evaluated the effect of progestin treatment on TDO2 expression in MM cells which always express wt-MED12 [4]. Cells obtained from eight unique patients were used and all treatments were completed in triplicate. Cells were each treated with increasing doses of R5020 and MPA. Figure 2B shows a dose-dependent decrease in TDO2 mRNA levels with increasing concentrations of R5020 (upper panel) and MPA (lower panel). R5020 at 10^–7^ M significantly decreased TDO2 expression with the peak reduction occurring at 10^–5^ M (0.40 ± 0.09 of control level, *P* < 0.003). TDO2 levels were decreased to 0.42 ± 0.03 of control level by 10^–8^ M MPA (*P* < 0.01), which further decreased to 0.16 ± 0.04 of control level at 10^–5^ M (*P* < 0.001). We performed a time course experiment and found that TDO2 gene expression in MM cells was inhibited by 10^–6^ M MPA at each time point (6, 24, 48, and 72 h) examined (*n* = 5, Supplemental Fig. 2). Sanger sequencing of genomic DNA isolated from tissue (data not shown) and cDNA isolated from treated cells of all 3 patients confirmed a pure wt-MED12 genotype (Supplemental Fig. 1B). These data suggest that progesterone inhibits TDO2 gene expression in LM and MM which express wt-MED12.

### MED12 Mutations Affect Progestin-Mediated TDO2 Gene Expression in G44D mut-MED12 Leiomyoma Cells

Next, we characterized whether MED12 mutation affects progestin-mediated TDO2 gene expression, which potentially contribute to the differential TDO2 expression observed in mut-MED12 LM vs. wt-MED12 LM. We assessed the dose response of G44D mut-MED12 LM cells subjected to the same treatment described above. Each experiment was done in triplicate and repeated in three unique patients. Figure 2C shows TDO2 mRNA levels after treatment with different doses of R5020 (upper panel) and MPA (lower panel) compared to vehicle. High doses of R5020 elicited a trend toward decreased TDO2 expression, but the effects did not reach statistical significance compared to vehicle controls (Fig. 2C, upper panel). MPA significantly decreased TDO2 mRNA levels starting at 10^–7^ M (0.43 ± 0.08 of control level, *P* < 0.005), but increases in dose did not further downregulate TDO2 expression (Fig. 2C, lower panel). Supplemental Fig. 1C shows the Sanger sequences for the treated cells from 3 patients. Note that each patient sample represents a mixture of wt-MED12 and G44D mut-MED12 cells.

**Fig. 3:**
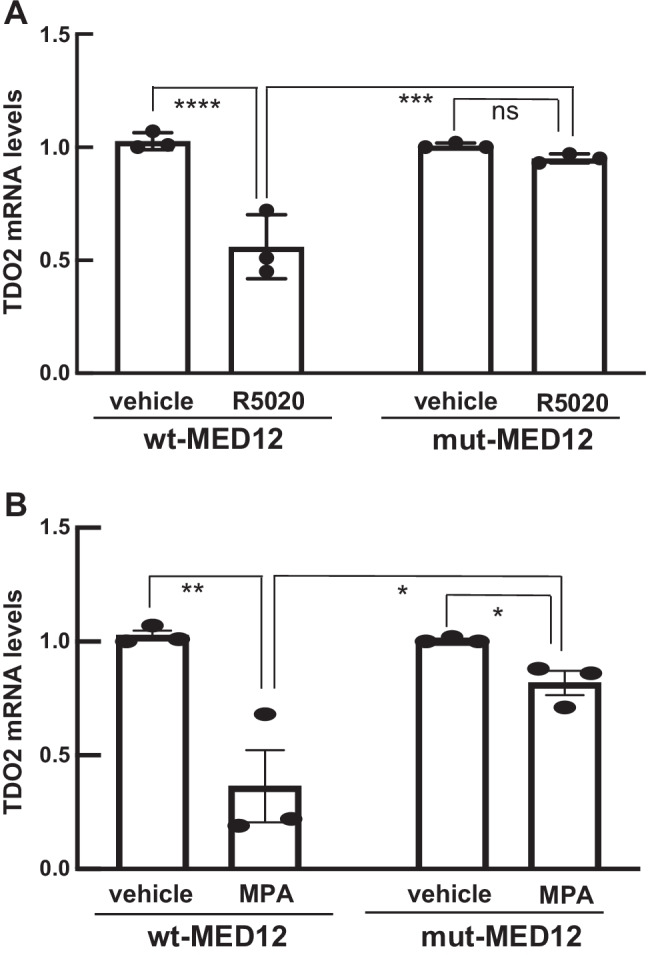
The inhibitory effect of progestin agonists on TDO2 gene expression is blunted in G44D mut-MED12 LM cells. Side-by-side comparison of TDO2 mRNA levels (real-time qPCR quantification) in first passage of G44D mut-MED12 LM cells treated with 10^–5^ M R5020 (**A**) or 10^–6^ M MPA (**B**) for 24 h with that in wt-MED12 LM. All values were normalized to TBP and compared to vehicle (0.1% ethanol). *P < 0.05, **P < 0.005, ***P < 0.001, ****P < 0.0005. ns: not significant

It has been reported that in vitro cell culture causes loss of mut-MED12 LM cells [16]; therefore, we treated first passage cells with 10^–5^ M R5020 or 10^–6^ M MPA based on the dose that elicited maximal downregulation of TDO2 expression in wt-MED12 LM cells (Fig. 2A). Each experiment was done in triplicate and repeated in three unique patients. Figure 3A shows that in G44D mut-MED12 LM cells, treatment with R5020 at 10^–5^ M had a minimal effect on TDO2 expression compared to vehicle-treated cells. Likewise, MPA at 10^–6^ M downregulated TDO2 mRNA level slightly but significantly in G44D mut-MED12 LM cells (0.82 ± 0.04 of control level, *P* < 0.05, Fig. 3B). For both progesterone agonists, the effect on TDO2 expression was blunted in mut-MED12 compared to wt-MED12 cells, which showed significantly reduced mRNA levels in response to both R5020 (Fig. 3A, P < 0.001) and MPA (Fig. 3B, P < 0.05). Sanger sequencing of cDNA from the treated cells from all 3 patients revealed a pure G44D mut-MED12 genotype. The sequences shown in Supplemental Fig. 1D are cropped to highlight the “hot-spot” of MED12 mutations at codon 44 in exon 2. These findings suggest that MED12 mutation decreases LM cells’ response to progestins, leading to a blunted downregulation of TDO2 gene expression that may account in part for the higher TDO2 expression and lower tryptophan levels in mut-MED12 LM cells.

## Discussion

In this study, we showed that TDO2 gene expression is upregulated in LM expressing mutated MED12, and that the regulatory effect of progestins (R5020 and MPA) on TDO2 expression in MM and wt-MED12 LM cells is lost or decreased in LM cells expressing G44D mut-MED12. These findings suggest that MED12 mutation may disturb progesterone signaling in LM that regulates TDO2 gene expression, leading to upregulated TDO2 gene expression and decreased tryptophan levels in mut-MED12 LM.

LM were once thought to have a single phenotype, but it has become clear that each fibroid represents a monoclonal tumor with a mutation signature, and focus has shifted to identifying common mutations and targeted approaches to prevention and treatment. In the past decade, a number of mutation groupings have been identified, the most common of which include mutations in MED12. Mut-MED12 has been identified in 70–75% of LM [4, 15, 17]. The MED12 encodes the mediator complex, which is highly conserved in eukaryotes and plays an important role in regulation of transcription through its interactions with specific transcription factors and RNA polymerase II; however, its role in fibroid growth and development has not been fully elucidated [4, 18]. LM harboring MED12 mutations have distinct transcription profiles, and candidate genes involved in LM tumorigenesis, such as IGF2 and WNT4, are specifically upregulated in mut-MED12 LM. The underlying mechanisms linking MED12 mutations to LM pathogenesis remain unclear and no clinically relevant therapies have been proposed to target specific mutation signatures [19–22]

Computational analyses have suggested that mut-MED12 in LM could have altered interactions with transcriptional co-activators; this idea is supported by findings that mut-MED12 disrupts the MED12-Cyclin C binding interface, leading to a loss of mediator-associated CDK activity [5, 23, 24]. Previously, we reported that mut-MED12 associates with PR at the chromatin level and the interactions between PR and chromatin are dysregulated in LM expressing the G44D mut-MED12 [25]. In this study, we found that progestins significantly inhibit TDO2 expression in MM and LM cells expressing wild-type MED12, and that MED12 mutation decreased the efficacy of this inhibitory effect, leading to upregulated TDO2 expression in mut-MED12 LM. It has been reported that TDO2 expression is higher in PR-negative versus PR-positive breast cancer tissues, suggesting that progesterone may inhibit TDO2 expression via PR in breast cancer cells [12]. Paradoxically, progesterone stimulates TDO2 expression in mouse uterine stroma cells [13]. The mechanisms underlying progesterone/PR-mediated target gene expression are complicated. PR exists as two isoforms, PR-A and PR-B, which may have distinct or similar functions depending on the promoter context and cell type [26–28]. In vitro cell culture model system which lack key in vivo conditions or cofactors may interfere with progesterone/PR signaling [29, 30]. Adding another layer of complex, glucocorticoids regulate TDO2 expression in a tissue-specific manner, and glucocorticoid receptor and PR share the same DNA-binding motif [31–33]. Given the important roles of tryptophan-kynurenine pathway in tumorigenesis, further studies are needed to clarify the effect of progesterone/PR signaling on TDO2 expression in LM using ex vivo tissue explant or in vivo xenograft mouse model.

Three enzymes (TDO2, IDO1, and IDO2) catalyze the first rate-limiting step of tryptophan metabolism through kynurenine pathway. We found that TDO2, but not IDO1/2, was upregulated in mut-MED12 LM, suggesting that TDO2 dysregulation may account for the reduced tryptophan levels in mut-MED12 LM subtype [6]. Soon after we submitted our manuscript, Chuang et al. also reported the aberrant overexpression of TDO2 expression in mut-MED12 LM [34]. TDO2 has been extensively studied in other organ systems and identified as an important factor in tumorigenesis and growth [7–10, 12]. Progesterone is critical to fibroid growth and anti-progestin therapies have shown promise in therapeutic management, but efficacy varies widely by patient, potentially due to variation in mutation status of treated fibroids [35–37]. Differential regulation of TDO2 in response to progesterone agonists in mut-MED12 LM versus wt-MED12 LM and MM may explain the variability in response to progesterone therapy and provide insight into therapeutic management of this specific LM subtype.

This study is limited by a small sample size and focus on a single candidate gene and a single MED12 mutation category, G44D. However, our novel finding that MED12 mutation in LM affects tryptophan metabolism is an important step toward defining the molecular and metabolic signatures of leiomyoma subgroups. By more completely understanding how varying genotypes affect hormonal response and regulation, we can more precisely target development of medical therapies. Continued research on this topic may identify targeted interventions for fibroids based on molecular signatures.

## Supplementary Information

Below is the link to the electronic supplementary material.

Supplementary file1 (PPTX 690 kb)

Supplementary file2 (DOCX 13 kb)

## Data Availability

Raw data were generated at Northwestern University. Derived data supporting the findings of this study are available from the corresponding author S.E.B. on request.
