# Supplementary material for: Tryptophan 2,3-Dioxygenase-2 in Uterine Leiomyoma: Dysregulation by MED12 Mutation Status
Source: Reprod Sci. 2022 Jan 21;29(3):743–9. doi: 10.1007/s43032-022-00852-y (PMC8863695; doi:10.1007/s43032-022-00852-y)
Supplement: Supplementary file 1 — Supplementary file1 (PPTX 690 kb) [file 43032_2022_852_MOESM1_ESM.pptx]

## Slide 1
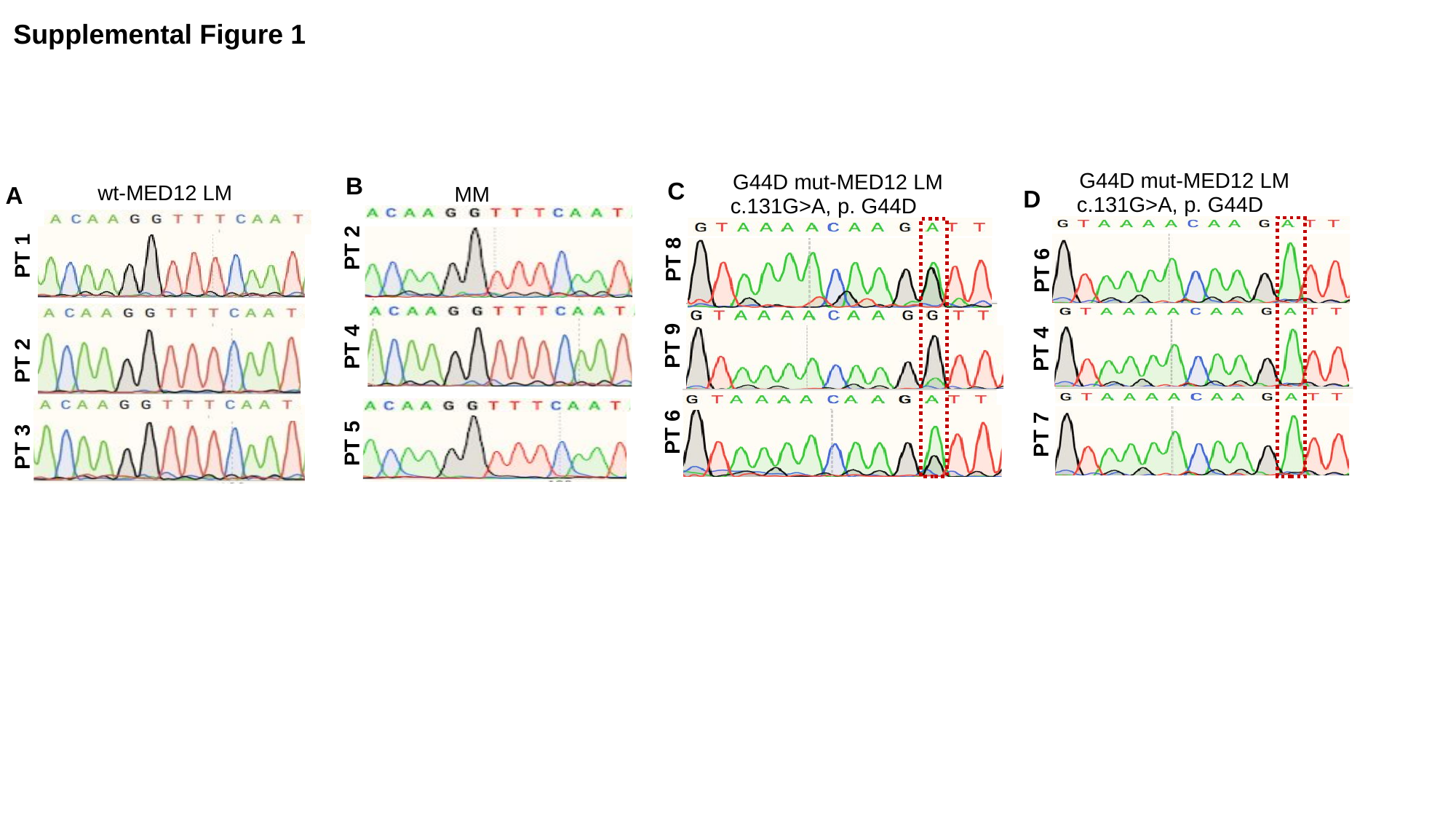

Supplemental Figure 1
G44D mut-MED12 LM
G44D mut-MED12 LM
B
C
wt-MED12 LM
A
MM
D
c.131G>A, p. G44D
c.131G>A, p. G44D
PT 8
PT 2
PT 6
PT 1
PT 4
PT 9
PT 4
PT 2
PT 7
PT 6
PT 5
PT 3

## Slide 2
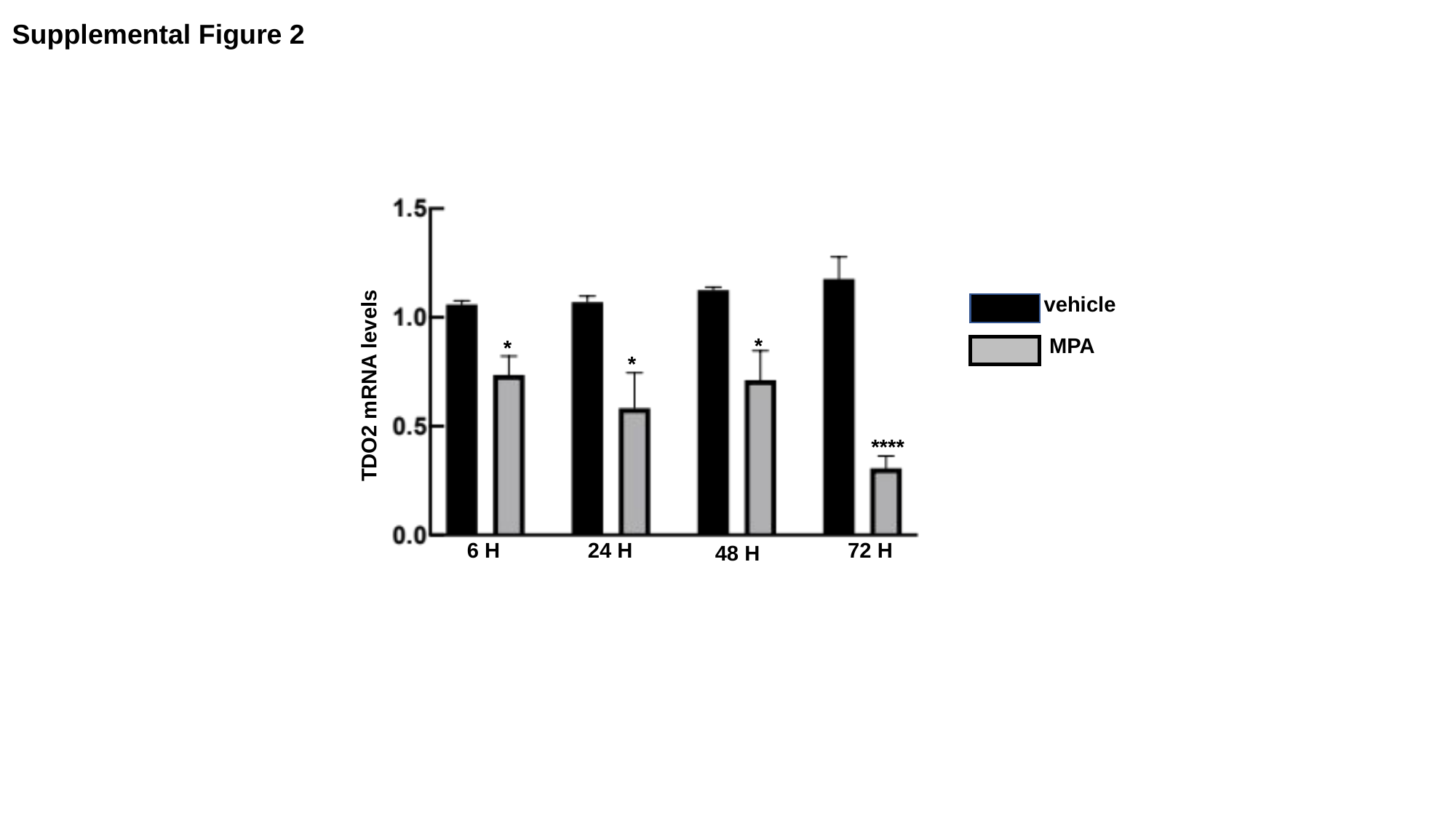

Supplemental Figure 2
vehicle
*
MPA
*
*
TDO2 mRNA levels
****
6 H
24 H
72 H
48 H
