# Supplementary material for: Tryptophan 2,3-Dioxygenase-2 in Uterine Leiomyoma: Dysregulation by MED12 Mutation Status
Source: Reprod Sci. 2022 Jan 21;29(3):743–9. doi: 10.1007/s43032-022-00852-y (PMC8863695; doi:10.1007/s43032-022-00852-y)
Supplement: Supplementary file 2 — Supplementary file2 (DOCX 13 kb) [file 43032_2022_852_MOESM2_ESM.docx]

**Supplemental Figure Legends**

**Supplemental Figure 1**: Sequence chromatograms showing Sanger sequencing data of cDNA isolated from treated cells. The sequences are cropped to highlight the “hot-spot” of MED12 mutations at codon 44 in exon 2. **A**: sequencing traces of wt-MED12 LM cells presented in Figure 2A, which had a pure wild-type MED12 genotype. **B**: sequencing traces of MM cells presented in Figure 2B, which had a pure wild-type MED12 genotype. **C**: sequencing traces of G44D mut-MED12 cells presented in Figure 2C, which showed a mixture of wt-MED12 and G44D mut-MED12 (c.131G>A, p.G44D) cells. **D**: sequencing traces of G44D mut-MED12 cells presented in Figure 3, which had a pure G44D mut-MED12 genotype (c.131G>A, p.G44D). PT, patient.

**Supplemental Figure 2: Effect of MPA treatment on TDO2 gene expression in primary MM cells.** Real-time qPCR quantification of TDO2 mRNA levels in primary cultured MM cells treated with vehicle (ethanol) or 10^-6^ M MPA for 6, 24, 48, or 72 h. All values were normalized to TBP and compared to vehicle. *P<0.05, *****P<0.0001*.
